# Reply to N. W. on Topical Cold in Gouty Inflammation

**Published:** 1815-11

**Authors:** Robert Kinglake


*September* 13, 1815.

---

*For the London Medical and Physical Journal.*

### *Reply to N. W. on Topical Cold in Gouty Inflammation;* by ROBERT KINGLAKE, M.D.

YOUR Langport correspondent under the anonymous designation of N. W. has adduced a case, in your last Number, of the effects of topical cold in rheumatic or gouty inflammation, precisely similar to that which popular prejudice and unfounded dread have long imagined must be almost the inevitable consequence of such treatment. The practice, however, has for several years been extensively pursued, as well under the direction of others as under my own immediate observation, without the vulgar and unintelligent fears of its baneful influence having, in a single instance, been warranted; and, I believe, not even reported,

 but but on one occasion before the present example. Alleged facts become of importance, first, by the internal evidence by which they may be accompanied; and, secondly, by the external testimony afforded them by concomitant circumstances. It will be seen, after bestowing a few observations on the recited case, how far it is entitled to credit on either the one or the other of these grounds of authority.

That an inflammatory affection of either the brain or of its membranes, called phrenitis, should result from subduing an acute degree of inflammation on the knee-joint, is by no means in the legitimate or reasonable order of cause and effect. If inflammatory action, situated in any part of the animal frame, be a condition of disease possessing a migrating quality, if it may station its violence at one hour on one part, and take its flight to another, however distant, at the next, the laws by which such a fugitive property is regulated are yet to be learnt and promulgated. The uninformed public in medical principles, and in the vital and motive resources of the animal economy, may imagine the most preposterous occurrences, the very grossest of all absurdities; and such crude and gratuitous notions may pass current amongst them; but N. W. will have much more difficulty in persuading the intelligent medical reader of the possibility of any thing of the kind to happen consistently with the immutable laws of animal life. To suppose, that an unexplained accident, such as has been recited by your correspondent, could be regarded as characteristic or demonstrative of the natural course of things, would be to be led sadly astray by a phantom of popular prejudice and irrational credulity. Your correspondent has, it must be owned, attired his solitary case in a dress well adapted to terrify the unthinking part of the public, and more particularly the female portion of the community, who are likely to be especially scared by the mysterious horrors of such a narrative; but the investigating medical reader will require your *initial* correspondent to state, much more minutely and correctly than he has done, all the relevant circumstances connected with the previous health of his phrenitic patient, the state of the general system when the inflammatory excitement on the knee-joint first made its appearance, whether an undigesting stomach, a constipated habit of bowels, or any other kind of visceral derangement either preceded or attended the sudden, but *instructive*, "translation" of morbid irritation from the knee to the brain, causing the *prophetic* sufferer to exclaim, "I am a dead man—I shall die—the pain has left my knee, and struck to my head !!!" Does your Langport correspondent conceive, that "translations" are as mechanical and direct

 in in the animal economy, as they are known to be in the *spiritual* offices of the church, and that, as in the latter case, they are performed by a *fiat*, by a *veritable congé d'elire*? Such proceedings are, indeed, unknown to Nature's laws, and of course are foreign to all that relates to animal life, health, and disease. The laws of sympathy are those of indivisible continuity and identity of vital power, and it is by the operation of these only in such circumstances that any correct idea can be formed of the origin, propagation, and establishment of disease. "N. W.'s objections and case" present a very common-place example of what the ancients denoted by the phrase of *inflammatory diathesis* pervading the whole system. It first shewed itself, it should seem, on the knee joint, the violence of which brought the prevailing disposition to a similar state of excitement into full action, and, finally, proved destructive by the irrecoverable injury inflicted on the nervous energy through the medium of the affected brain. The topical cold to which this eventual mischief has been imputed, had a tendency to prevent rather than to induce the alleged affection of the brain. The inflammatory action at the joint gradually extended its diseased influence over the system, and that too in the exact ratio of its violence; it, therefore, was the direct and efficient cause of all the morbid derangement that discovered itself, either on the brain or elsewhere. The cessation then of this *cause*, which resulted from the use of topical cold, could not have increased the pernicious *effect*! It would, indeed, be at utter variance with common sense, and a solecism in reasoning, to contend, that the abstraction of a cause must be the augmentation of its effect! *Sublata causa tollitur effectus*, is too obviously and undeniably true to be resisted by any one who may be either capable of reasoning, or who may be inclined to prefer correctness to error.

This *knee-vanishing* and *brain-killing* case of your correspondent has been deplorably misunderstood. The *knee* part of this mortal disease seems to have formed but a very inconsiderable ingredient in the general affection. It presented a morbid feature only of the inflammatory diathesis then pervading the system. If all had been well on the part of the general frame; if the local affection had been the whole of the disease; its extinction, however affected, whether by cold water or by spontaneous dispersion, would have proved curative; but, that not being the case, the system at large being in fault, the conflict was with the *whole* and not with a *part* only.

It appears allowable to reason thus on the merits of the Langport case, as, in the great majority of instances in which

 the the cooling treatment has been employed, the removal of the local inflammation has proved a speedy and a permanent cure. No *translations* nor transfers of the disease have been made to any visceral part. In general, the inflammatory diathesis in the system either does not exist at all, or in so small a degree as not to be sufficiently affected by the local pain and inflammation in gouty disease, to induce a serious extent of visceral excitement. The uninterrupted continuity of vital power renders the system, at all times, liable to be more or less affected by local irritation; but, in these instances, and particularly with reference to gout, a salutary restraint will be imposed on that liability, by a prompt and an effectual removal of the local pain which may have excited it, and which, if prolonged, may formidably increase it.

Phrenitis, under any circumstances, is a disease fraught with the utmost danger to life; its very existence, however short its duration, must have resulted from a derangement of the healthful state of the brain that might be absolutely irreparable; on the contrary, if the injury has been less hurtful, recovery might follow. Your correspondent's case, therefore, probably would not have terminated favourably, if it had presented to him in its naked simplicity, unobserved by the delusive anomaly of an inflamed knee. Indeed, to have afforded any chance of success in such a case, bleeding should have been carried incomparably further than appeared to your correspondent to have been necessary. Phrenitis, with the vascular "throbbing" and fullness described, should have been combated with bleedings to the extent of at least eighteen ounces *every six hours*, as long as such imperative indications required them. But here again, that appalling hobgoblin, the dread of *repelled* gout, entered its fearful *caveat* against weakening the powers of life too much by depletion. Not to do enough on these occasions is worse than doing nothing at all; the object to be effected then remains unaccomplished, and grounds are laid for a subsequent cause of erroneous reasoning and pernicious practice. Your correspondent's idea of reducing the powers of life, by diminishing the contents of the sanguiferous system, and their circulating impetus, previously to employing topical cold in gouty disease, lest the repelled action should precipitate the accelerated current on some vital part, is more applicable to the mechanical laws of *hydraulics* than to those by which the animal machine is governed and actuated. N.W. should not have perplexed himself with the abstruseness of a physiological question if he did not know how to discuss it on its own legitimate grounds. The laws of *vital* action have no affinity to those of *lifeless* mechanism; nor does your correspondent appear to perceive, that, by going beyond the reach of his comprehension, he has virtually vindicated the very treatment he has so unsparingly condemned. He speaks of "precautionary measures," of vascular depletion, and of unfitting the system for dangerously hurrying the circulating fluids on vital organs. By these measures, the general powers of the system, in an inflammatory disease, might, no doubt, be salutarily diminished, the liability to hurtful excitement in any part of the frame might be lessened, and thus an exemption might be obtained from the injurious consequences of such an affection. But does not the influence of topical cold in local inflammation accomplish the same much more directly and much more effectually? Does it not remove the chief cause of the systematic derangement? Does it not abstract diseased excitement, and thereby prevent inordinate action from being propagated to the system? If the local disease be subdued, where is the remaining active source of mischief to the system? A suitable diminution of the high tone and plenitude of health has always been considered as an essential auxiliary to the curative influence of topical cold in obstinate cases of gouty inflammation, and that solely for the purpose of obviating the possible mischief of inflammatory excitement, and in no wise to provide against the groundless dread of the local disease being transferred to the system in a state of high susceptibility for carrying it to a destructive length of violence! The systematic power which your correspondent would diminish by his "precautionary measures," has been supposed, by the adversaries of the cooling treatment of gout, to be indispensably necessary, first to reject, and afterwards to establish, the gouty excitement on the affected joint. In ordinary cases, topical cold, in gouty inflammation, is fully sufficient to subdue the inflammatory action, without requiring the co-operative aid of reducing the strength of the system; but, where the influence of the local disease may have excited symptoms of general irritation, the assistance of collateral help may be requisite. But, with regard to protecting the system from an *ideal* retrocession of gouty disease, your correspondent is wrong in preparing his "precautionary measures." By debilitating the systematic strength, the power of resistance would be diminished; and, by destroying the tone and fixedness of health, a state of irritability would be induced exceedingly favourable to the general diffusion, as well as to a visceral preponderance, of disease. By subduing gouty inflammation on the affected part, a noxious stimulus to the system is removed; and to suppose that the negation or abstraction of a power is its positive exertion, is a strange contradiction contradiction in terms, a deplorable inversion of the natural order of things; and to imagine that such a state of comparative inaction should have an exciting cause of injury to the system, is certainly the most far-fetched and the most untenable of all silly apprehensions.

The defect of internal evidence of the truth of the case recited, will be apparent in the observations that have been offered. As to the external testimony, I may be justly allowed to ask your correspondent, why he has preferred the expedient of *initials*, to a full disclosure of his signature? The subject is too grave a one to be discussed without the authority of a name. The name and abode of the patient also should, on such an occasion, have been freely given, that a practicable clue might have been afforded for ascertaining various circumstances relating to the case which have been withheld; and, without which, it is impossible for the loose and meagre allegations contained in the recital duly to authenticate it. Numerous are the instances, (too much so indeed for me to recollect the amount of them,) in which the voice of vague rumour and the reports of hear-say officiousness, have cited cases of the destructive effect of topical cold in gouty inflammation, more terrific even than that of your correspondent; but, upon enquiry, not one of them has been verified, nay, the investigation has only served to detect, refute, and expose, many *maliciously* invented, as well as *credulously* adopted, calumnies against the cooling treatment of gout.

Your correspondent says, that he believes there is not a "solitary individual" in the medical profession who agrees with me in opinion respecting the extent to which I have proposed the cooling treatment of gout. By *lapse* rather than by design, he is quite correct in saying, there is not "a solitary individual;" far from it, indeed, a very large number ("not a solitary individual,") of advocates of the treatment are daily practising it as advised by me, with every gratification that a direct, safe, and permanent remedy can afford them. This affirmation is made *meo periculo* with respect to its correctness, and has already, and may again be, substantiated by reference to an ample stock of written assurances addressed to me of the fact.

Improvement in medical practice can never be effected by *anonymous garblings* of cases. Scientific researches should be conducted in a very different spirit; the desire of truth should be the master-feeling and the unceasing object of pursuit, and should be sought after at least with liberality and candour, and, if possible, with adequate intelligence. To notice an anonymous communication on a medical subject might might be justly regarded as *infra dignitatem;* and, in that sentiment, I should have been silent on the present occasion, if the importance of the subject attached did not require that it should be defended from so unworthy a mode of assailing it, and more particularly from its having been done in the immediate sphere of my professional practice, with probable views that it would not become me to arraign, and which I shall, therefore, leave exclusively to the correction of the author's own reflections.

Nil adeo fieri celeri ratione videtur
Quam si mens fieri proponit, et inchoat ipsa,
Ocius ergo animus quam res se perciet ulla,
Ante oculos quarum in promptu natura videtur. LUCR.

*Taunton;*
*September* 13, 1815.

